# Supplementary material for: Endothelium-specific depletion of LRP1 improves glucose homeostasis through inducing osteocalcin
Source: Nat Commun. 2021 Sep 6;12:5296. doi: 10.1038/s41467-021-25673-6 (PMC8421392; doi:10.1038/s41467-021-25673-6)
Supplement: Supplementary file 1 — Supplementary Information [file 41467_2021_25673_MOESM1_ESM.pdf]

SUPPLEMENTARY INFORMATION

Supplementary Table 1. Representative genes that are regulated in ECs isolated from LRP1 eKO mice, analyzed with mRNA sequencing.

Gene Symbol	Fold change (log2)	
	MHLEC	MLivEC
alox5ap	2.15679311	1.938323017
ocn2	4.60029667	6.087391462
ocn1 (ocn)	4.91337619	5.861306677
selplg	3.67567119	1.872018864
timp4	-2.1184809	-1.750636851
hmgcs2	-2.2955471	-1.4651599
lrp1	-4.9463174	-3.621346872

Supplementary Table 2. Sequence information for qPCR primers

**Gene**	Forward primer	Reverse primer
LRP1	5'- ggaccaccatcgtggaaa-3'	5'-tcccagccacggtgatag-3
Selplg	5'- tctggcagtgtggactgg-3'	5'- caaggaagcttggggacat-3'
Alox5ap	5'- catgaaagcaaggcgcata-3'	5'- cgcatctacgcagttctgg-3'
OCN (recognizing both OCN1&2)	5'- agactccggcgctacctt-3'	5'- ctcgtcacaagcagggttaag-3'
OCN1	5’- cttggcccagacctag-3’	5’- gctgggcttggcatctg-3’
OCN2	5’- cttggtgcacacctag-3’	5’- gctgggcttggcatctg-3’
HMGCS2	5'- aggatgcttccccaggtt-3'	5'- gagtctagacgtttgggcaga-3'
Timp4	5'- agggagagcctgaatcatca-3'	5'- gcactgcatagcaagtggtg-3'
GAPDH	5'- tgtccgtcgtggatctgac-3'	5'- cctgcttcaccaccttcttg-3'

Supplementary Table 3. Key resource table

**REAGENT or RESOURCE**	**SOURCE**	**IDENTIFIER**
**Antibodies**		
LRP1-CTD, 1:1000	Sigma	Cat#L2170
LRP1 (8G1), 1:50	Abcam	Cat#ab20384
PECAM-1 (CD31), 1:1000	BD Biosciences	Cat#553369
Osteocalcin, 1:200	Santa Cruz	Cat#sc-376726
IRS-1, 1:500	Cell signaling	Cat#2382
Phospho- IRS1 (Tyr612), 1:500	Millipore	Cat#09-432
Akt, 1:1000	Cell signaling	Cat#9272
phospho-Akt (Ser473), 1:1000	Cell signaling	Cat#9271
Phospho-Akt (Thr308) (D25E6), 1:1000	Cell signaling	Cat#13038
GSK-3β (D5C5Z), 1:1000	Cell signaling	Cat#12456

Phospho-GSK-3β (Ser9) (D85E12), 1:1000	Cell signaling	Cat#5558
Rabbit polyclonal anti-Glut4, 1:500	EMD Millipore	Cat#07-1404
Phospho-IGFI Receptor β (Tyr1135/1136)/Insulin Receptor β (Tyr1150/1151) (19H7), 1:1000	Cell signaling	Cat#3024
FOXO1, 1:1000	Cell signaling	Cat#2880
FOXO3A, 1:500	Thermo Scientific	Cat#MA5-14932
FOXO4, 1:1000	Cell signaling	Cat#9472
IGF1R (Ab-1161), 1:500	Sigma-Aldrich	Cat#SAB4300359
GPRC6A, 1:500	Sigma-Aldrich	Cat#SAB4500879
V5 Epitope Tag Antibody, HRP conjugate, 1:1000	Thermo Scientific	Cat#R960-25
β-Actin (C4) -HRP, 1:2000	Santa Cruz	Cat#sc-47778
Anti-Flag antibody, 1:1000	Sigma-Aldrich	Cat#H7425
**Chemicals, Peptides, and Recombinant Proteins**		
EZview Red ANTI-FLAG M2 Affinity Gel	Sigma-Aldrich	Cat#F2426
Glutathione agarose	Thermo Scientific	Cat#16100
60% high-fat diet	Research Diets	Cat#D12492
Streptozotocin	Sigma-Aldrich	Cat#S0130
Insulin	Novolin R	Cat#0169-1833-11
Osteocalcin (OCN) protein	Abcam	Cat#152231
Protein A/G Plus-agarose beads	Santa Cruz	Cat#sc-2003
iScript™ cDNA synthesis kit	Bio-Rad Laboratories	Cat#1708891
iTaq SYBR Green supermix	Bio-Rad Laboratories	Cat#1725121
TaqMan™ Universal PCR Master Mix	Thermo Scientific	Cat#4304437
Insulin solution	Santa Cruz	Cat# 11061-68-0
Insulin-like Growth Factor-I	Sigma-Aldrich	Cat#I3769
Tamoxifen	Sigma-Aldrich	Cat#T5648
Osteocalcin shRNA (m) lentiviral Particles	Santa Cruz	Cat#sc-40791-V
IGF1R siRNA (m)	Santa Cruz	Cat#sc-35638
Lipofectamine 2000	Thermo Scientific	Cat#11668019
Lipofectamine LTX with PLUS	Thermo Scientific	Cat#15338100
Perfusion Medium	Thermo Scientific	Cat#17701038
Collagenase	Sigma-Aldrich	Cat#C5168
Collagenase I	Worthington	Cat#LS004196
Collagenase II	Worthington	Cat#LS004174
DMEM	Thermo Scientific	Cat#11965118
William's E Medium	Thermo Scientific	Cat#12551-032
Wash Medium	Thermo Scientific	Cat#17704-024
Percoll	GE Healthcare	Cat#17089101
Penicillin & Streptomycin	Thermo Scientific	Cat#15140122
MCDB131	Sigma-Aldrich	Cat#M8537
Fetal bovine serum (FBS)	Sigma-Aldrich	Cat#F0926
Horse serum	Thermo Scientific	Cat#16050114
Hydrocortisone	Sigma-Aldrich	Cat#H0888

VEGF165	Sigma-Aldrich	Cat#V5765
Maintenance Supplements	Thermo Scientific	Cat#CM4000
Geneticin	Thermo Scientific	Cat#10131035
Calcein	Sigma-Aldrich	Cat#C0875
DSP	Thermo Scientific	Cat#22585
**Critical Commercial Assays**		
Insulin ELISA kit	Millipore	Cat#EZRMI-13K
Glucose uptake kit (colorimetric)	Abcam	Cat#ab136955
Plasma FFA assay kit	WAKO Chemicals	Cat#999-34691
Infinity Triglyceride kit	Thermo Scientific	Cat#TR22421
RNA purification kit	QIAGEN	Cat#74104
Glucose Uptake-Glo™ Assay Kit	Promega	Cat#J1341
Infinity Glucose kit	Thermo Scientific	Cat#TR15421
Human osteocalcin ELISA kit	Abcam	Cat#AB1952141
Mouse osteocalcin ELISA kit	Quidel	Cat# 60-1305
Mouse Glu-Osteocalcin High Sensitive EIA Kit	Takara Bio	MK129
Mouse Glu-Osteocalcin High Sensitive EIA Kit	Takara Bio	MK127
**Experimental Models: Cell Lines**		
Primary hepatocytes	This paper	N/A
Primary mouse lung and heart endothelial cells	This paper	N/A
Primary mouse liver endothelial cells	This paper	N/A
Primary mouse lung endothelial cells	Cell Biologics	Cat#C576011
C2C12	ATCC	Cat#CRL-1772
HEK293 cells	ATCC	Cat#CRL-1573
E.coli Lemo21 (DE3)	Biolabs	Cat#C2528J
**Experimental Models: Organisms/Strains**		
Mouse: B6;129S7-*Lrp1*^{tm2Her}/J (LRP1-floxed; LRP1f/f)	Jackson Laboratories	Cat#012604
Mouse: Cdh5(PAC)-CreERT2 (Cdh5-CreER+)	Dr. Ralf H. Adams from Max Planck Institute for Molecular Biomedicine, Germany	N/A
Mouse: WT C57Bl6/J	Jackson Laboratories	RRID: IMSR_JAX:000664
Mouse: B6;129-*Igf1r*^{tm2Arge}/J (IGF1R-floxed; IGF1Rf/f)	Jackson Laboratories	Cat#012251
**Deposited Data**		
Raw and analyzed RNA sequencing data from ECs isolated from LRP1 eKO and their littermate control mice	This paper	GEO: GSE117560
**Recombinant DNA**		
pCMV-Tag2 vector-LRP1β	This paper	N/A
pCMV-Tag2 vector	Agilent	Cat#211172

Recombinant adeno-associated virus expressing mouse osteocalcin shRNA	VectorBuilder	N/A
pLenti-C-Myc-DDK-OCN	This paper	N/A
pcDNA3 Flag FKHR AAA mutant (CA-FoxO1)	Addgene	Cat#13508
IGF1R-pLX307	Addgene	Cat#98344
Ad-CMV-iCre	Vector Biolabs	Cat#1045
**Software and Algorithms**		
Graphpad Prism for statistical analysis	GraphPad	N/A
ImageJ	NIH	https://imagej.nih.gov/ij/
**Other**		
MasterFlex pump	Cole-Parmer	Cat#Model77122-24

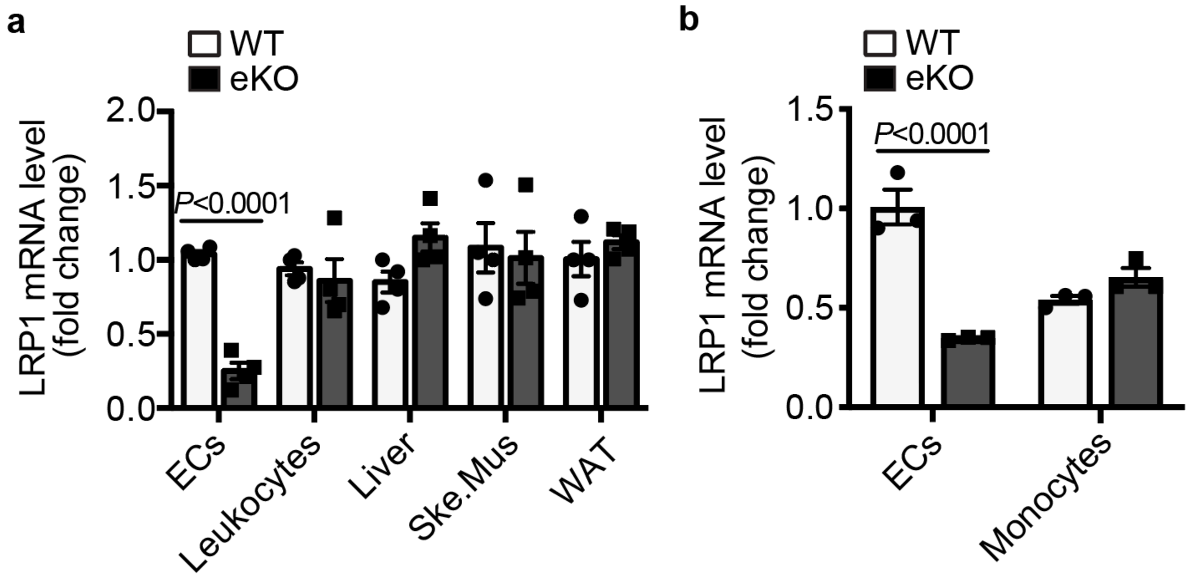

Supplementary Figure 1. LRP1 is specifically depleted in ECs. Real-time PCR assays for LRP1 were performed with indicated cells and tissues (**a-b**). eKO, LRP1$^{f/f}$; Cdh5-CreER$^{+/-}$. WT, LRP1$^{f/f}$; Cdh5-CreER$^{-/-}$. EC, endothelial cell. Ske. Mus., skeletal muscle. WAT, white adipose tissue. n=4 (**a**) and 3 (**b**). Data are presented as mean ± SEM. Analysis was one-way ANOVA followed by Fisher's LSD multiple comparison test (**a**) or unpaired two-tailed Student's *t* test (**b**).

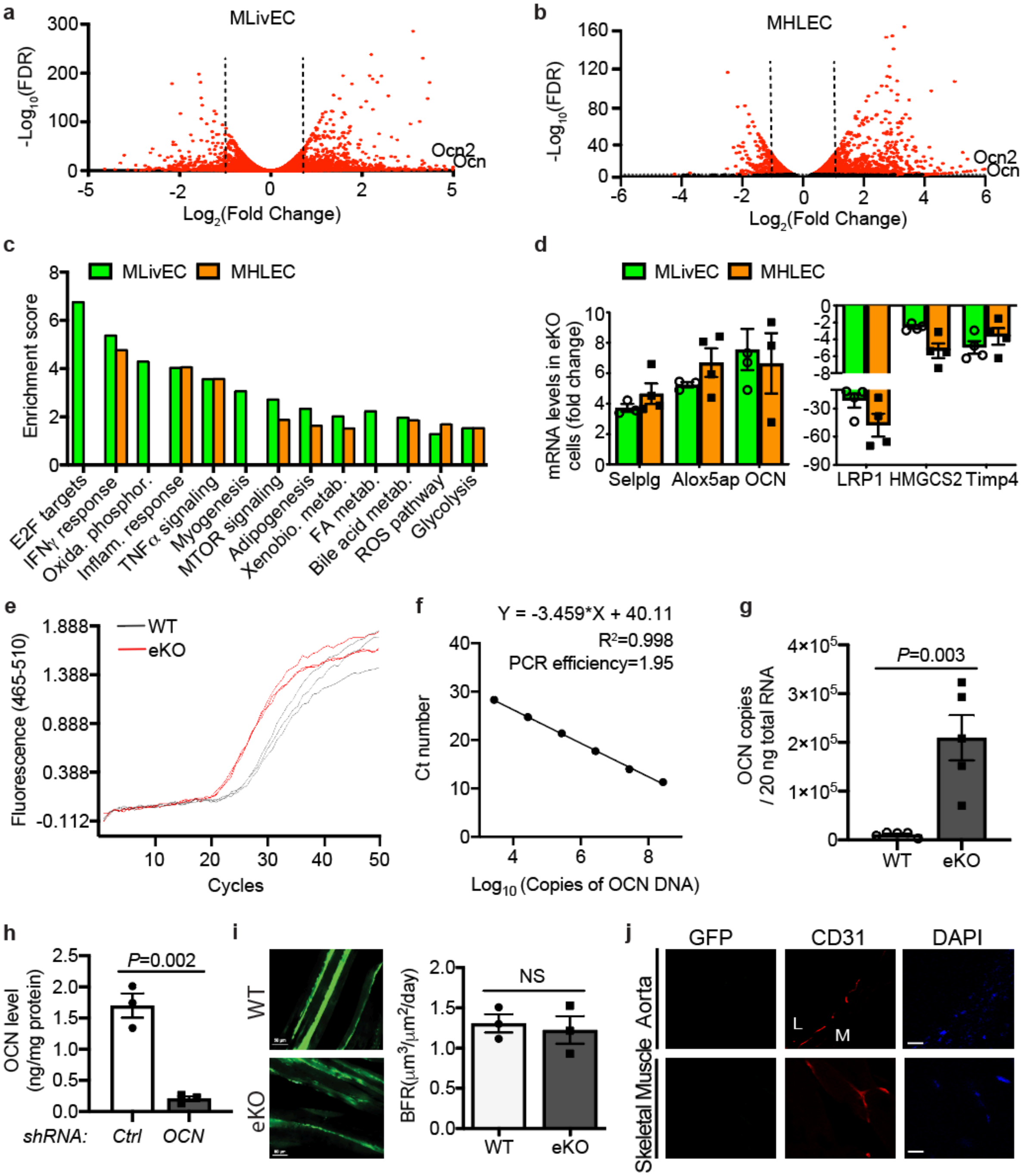

Supplementary Figure 2. Gene expression profiling analysis, circulating OCN level with LRP1-depleted ECs. (a-b) Volcano plots of mRNA-seq data for MLivECs (**a**) and MHLECs (**b**). (**c**) Normalized enrichment scores show changes of multiple pathways in LRP1-depleted MLivECs and MHLECs compared to WT ECs. (**d**) Representative gene expression changes identified in mRNA-seq data, presented as fold changes in LRP1-depleted ECs compared to WT ECs. (**e**) Representative qPCR amplication curves for OCN in WT and LRP1-depleted MHLECs. (**f**) The standard curve established between log of OCN DNA copies vs. cycle threshold (Ct)

obtained using 10-fold serial dilution of OCN plasmid DNAs. (**g**) OCN copies in WT and LRP1 eKO MHLECs were calculated based on its standard curve in (**f**). (**h**) OCN levels was markedly decreased in CM collected from mouse lung ECs transfected with OCN siRNAs, compared to control cells. This data validated our OCN ELISA assays. (**i**) Bone formation rates (BFRs) in LRP1 eKO and WT mice. Bone formation was visualized by double calcein labeling in the vertebrae. scale bar: 50 μm. (**j**) Negative control images for Figure 2b. Cross-sections of aorta and skeletal muscle in non-transgenic control mice were stained for GFP (green), CD31 (red) and DAPI (blue). L, lumen. M, media. Scale bar, 20 μm. n=3 (**d,** selplg, Alox5ap, OCN; **h**, **i**), 4 (**d,** LRP1, HMGCS2, Timp4), 5 (**g**). NS, not significant. Data are presented as mean ± SEM. Analysis was unpaired two-tailed Student's *t* test (**g-i**).

a

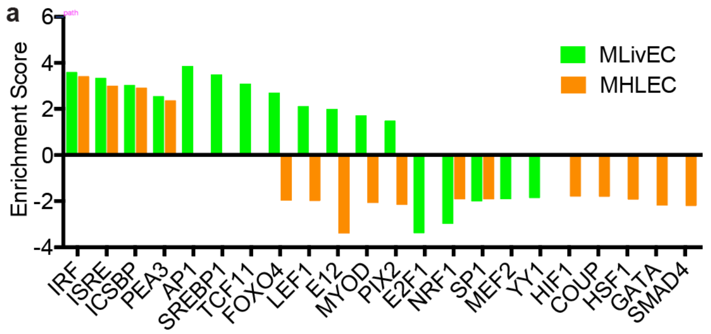

b

TF	*p*-value	#Genes in universe	#Genes in dataset	Target gene examples
Foxo4	0.002	45956	2559	IL7R,SEMA3A,IFIH1,GHR
Foxo1	0.014	45956	485	PDGFA,MAP4K5,MMP13
Foxo3	0.040	45956	245	SEMA3A,RARB,ID2,BDNF

c **d**

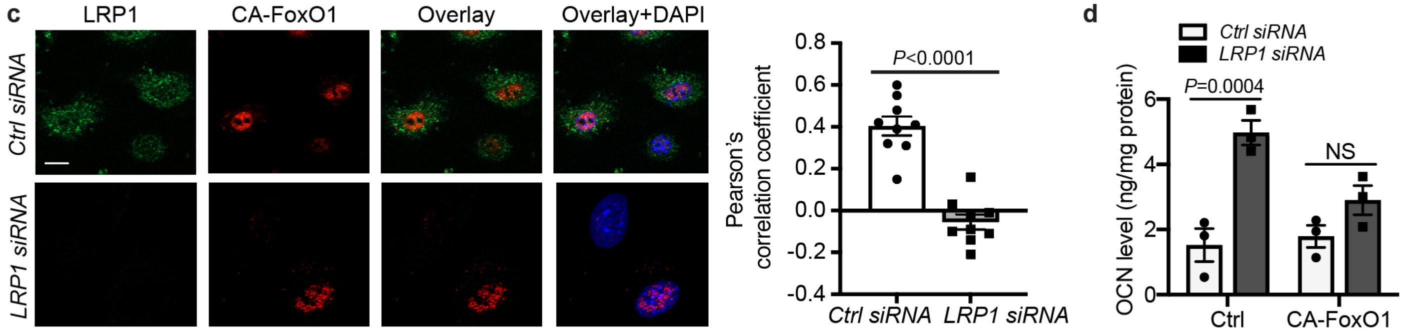

Supplementary Figure 3. FoxO1 is required for LRP1 depletion-induced OCN in ECs. (**a**) Normalized enrichment scores show changes of potential transcriptional factors (TFs) in LRP1-depleted MLivECs and MHLECs compared to WT ECs. (**b**) GESA analysis indicates that numbers of FoxO-responsive genes that are potentially regulated in LRP1-depleted ECs. (**c**) The expression and nuclear localization of constitutive active FoxO1 (CA-FoxO1) following the transfection of CA-FoxO1 and LRP1 siRNAs in MVECs. The co-localization of LRP1 and CA-FoxO1 was presented with Pearson's correlation coefficients. scale bar, 10 μm. (**d**) Constitutively active FoxO1 (CA-FoxO1) inhibited LRP1 depletion-induced OCN, analyzed with ELISA. n=9 (**c**), 3 (**d**). NS, not significant. Data are presented as mean ± SEM. Analysis was unpaired two-tailed Student's *t* test (**c**) or two-way ANOVA followed by Fisher's LSD multiple comparison test (**d**).

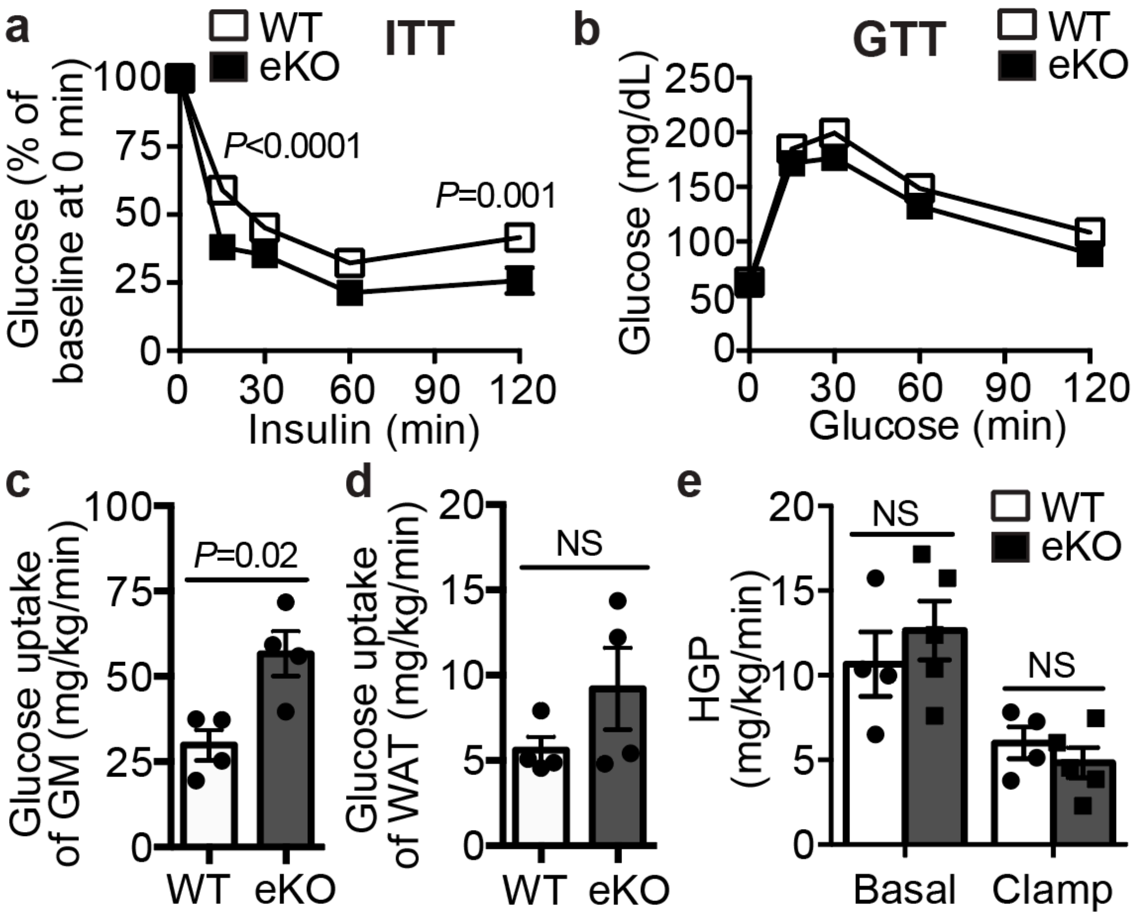

Supplementary Figure 4. LRP1 eKO mice display increased glucose uptake at physiological condition. (**a-b**) Insulin (**a**) and glucose (**b**) tolerance tests (ITTs, GTTs). (**c-e**) Hyperinsulinemic-euglycemic glucose clamp studies were performed in WT and LRP1 eKO mice for measurements of glucose uptake in gastrocnemius muscle (GM, **c**) and white adipose tissue (WAT, **d**) and hepatic glucose production (HGP, **e**). n=7 (**a**, WT), 6 (**a**, eKO), 9 (**b**), 4 (**c, d, e**, WT), 5 (**e**, eKO). NS, not significant. Data are presented as mean ± SEM. Analysis was two-way ANOVA followed by Fisher's LSD multiple comparison test (**a, b, e**) or unpaired two-tailed Student's *t* test (**c, d**).

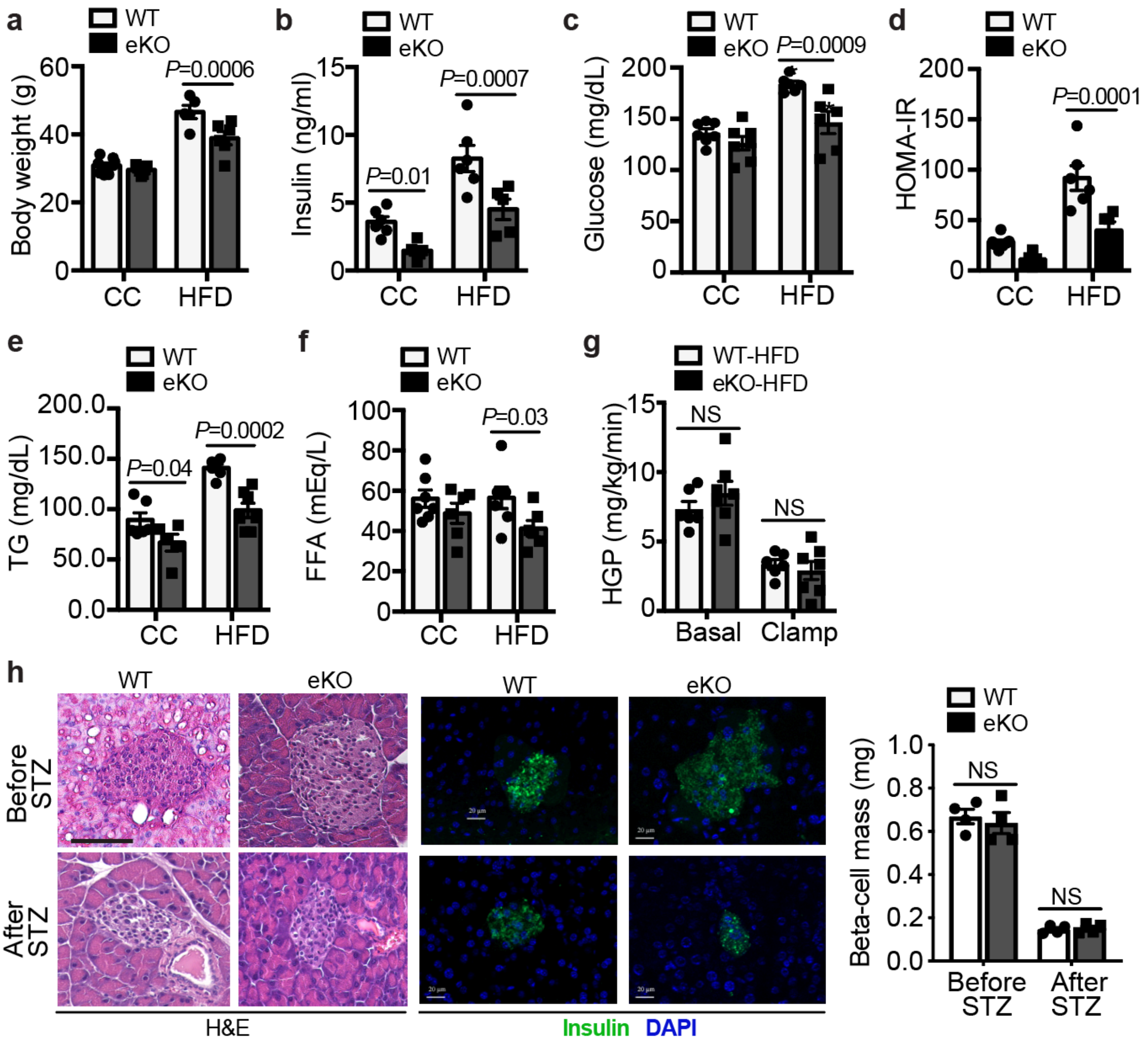

Supplementary Figure 5. LRP1 eKO mice display improved lipid and glucose responses after HFD feeding. (**a**) Body weight changes upon HFD feeding. CC, control chow. HFD, high-fat diet. (**b**) Blood insulin levels. (**c**) Blood glucose levels. (**d**) HOMA-IR scores. (**e**) Blood TG levels. (**f**) Blood FFA levels. (**g**) Hyperinsulinemic-euglycemic glucose clamp studies were performed in WT and LRP1 eKO mice fed HFD for measurements of hepatic glucose production (HGP). (**h**) H&E and insulin immunostaining were performed with pancreatic sections of RP1 eKO and WT mice before and after STZ treatments and beta-cell masses were estimated. Scale bar, 100 µm (H&E) and 20 µm (insulin images). n=7 (**a-d**, **f**, WT), 6 (**a-d**, **f**, eKO; **e-g**, WT), 5 (**e-g**, eKO), 4 (**h**). NS, not significant. Data are presented as mean ± SEM. Analysis was two-way ANOVA followed by Fisher's LSD multiple comparison test (**a-h**).

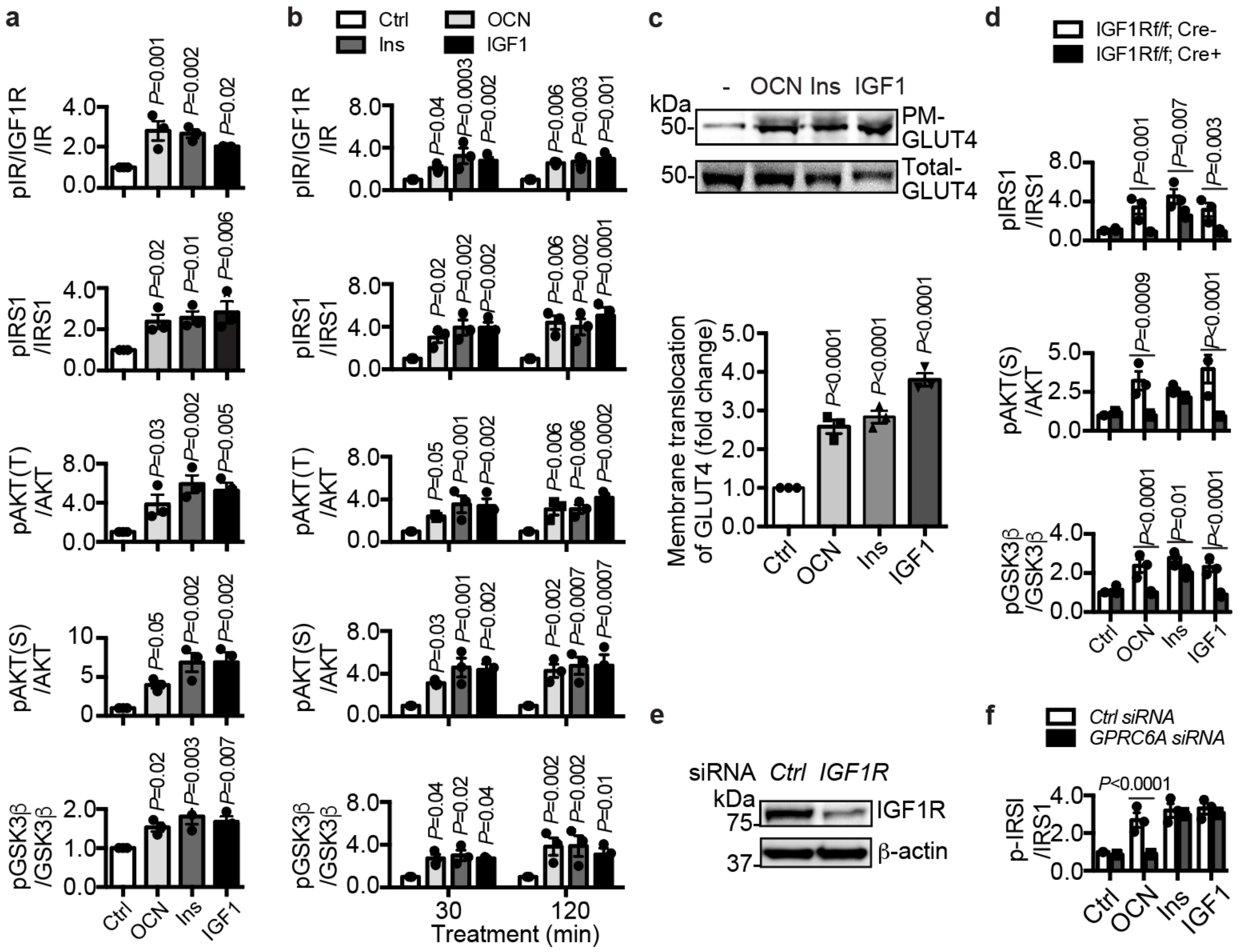

Supplementary Figure 6. OCN transactivates the insulin signaling pathway. (**a-b**) Quantitative results for Figure 5a (**a**) and 5b (**b**). (**c**) OCN increases membrane translocation of GLUT4 in skeletal muscle. (**d**) Quantitative results for Figure 5h. (**e**) The knockdown of IGF1R by its siRNAs in C2C12 myoblasts. (**f**) Quantitative results for Figure 5j. n=3 (**a-d, f**). Data are presented as mean ± SEM. Analysis was one-way ANOVA(**a**, **c**) or two-way ANOVA(**b**, **d**, **f**) followed by Fisher's LSD multiple comparison test.
